# An effective method for *Agrobacterium tumefaciens*-mediated transformation of *Jatropha curcas* L. using cotyledon explants

**DOI:** 10.1080/21655979.2020.1831363

**Published:** 2020-10-19

**Authors:** Ying Liu, Xiaoyan Yang, Yahuan Zhao, Yuesheng Yang, Zhenlan Liu

**Affiliations:** a State Key Laboratory for Conservation and Utilization of Subtropical Agro-bioresources, College of Life Sciences, South China Agricultural University, Guangzhou, China; b College of Coastal Agricultural Sciences, Guangdong Ocean University, Zhanjiang, China; c Department of Park, Yantai Kunyu Mountain Forest Station, Yantai, China

**Keywords:** Cotyledon explants, *Jatropha curcas* L., bud regeneration, Thidiazuron, transformation

## Abstract

Jatropha curcas is one of oilseed crops and has been considered as an energy crop. In the present study, efficient plant regeneration protocol and transformation method were developed for *J. curcas*. Because the regeneration efficiency of adventitious bud from cotyledon explants of *J. curcas* induced by traditional methods is low, and it takes a long time to get complete plants. It is necessary to establish a new regeneration system to improve regeneration efficiency. Cotyledon explants were dipped into TDZ solution at different concentrations respectively for various times to obtain higher efficiency of adventitious bud regeneration. This new regeneration method was then applied to genetic transformation of *J. curcas*. Cotyledon explants were precultured for 1 day after treated with high concentration of Thidiazuron (TDZ) solution (20 mg/L for 40 min), followed by *Agrobacterium tumefaciens* infection. After co-cultured for 2 days, the explants were placed on the induction hormone-free media for bud regeneration and resistant screening. After 30 days, selected shoot buds were transferred onto elongation medium for 15 days. Young leaf sections of the regenerated shoots were used for PCR (Polymerase chain reaction) detection of the transgenic shoots. The PCR positive shoots were isolated and used for *in vitro* grafting. The intact plants were obtained within 20 days. GUS (β-Glucosidase) staining and Southern analysis confirmed the transformation events. Briefly, a transformation efficiency of 34.32% was achieved and an intact transgenic plant could be obtained within 65 days.

## GRAPHICAL ABSTRACT

**Figure:**
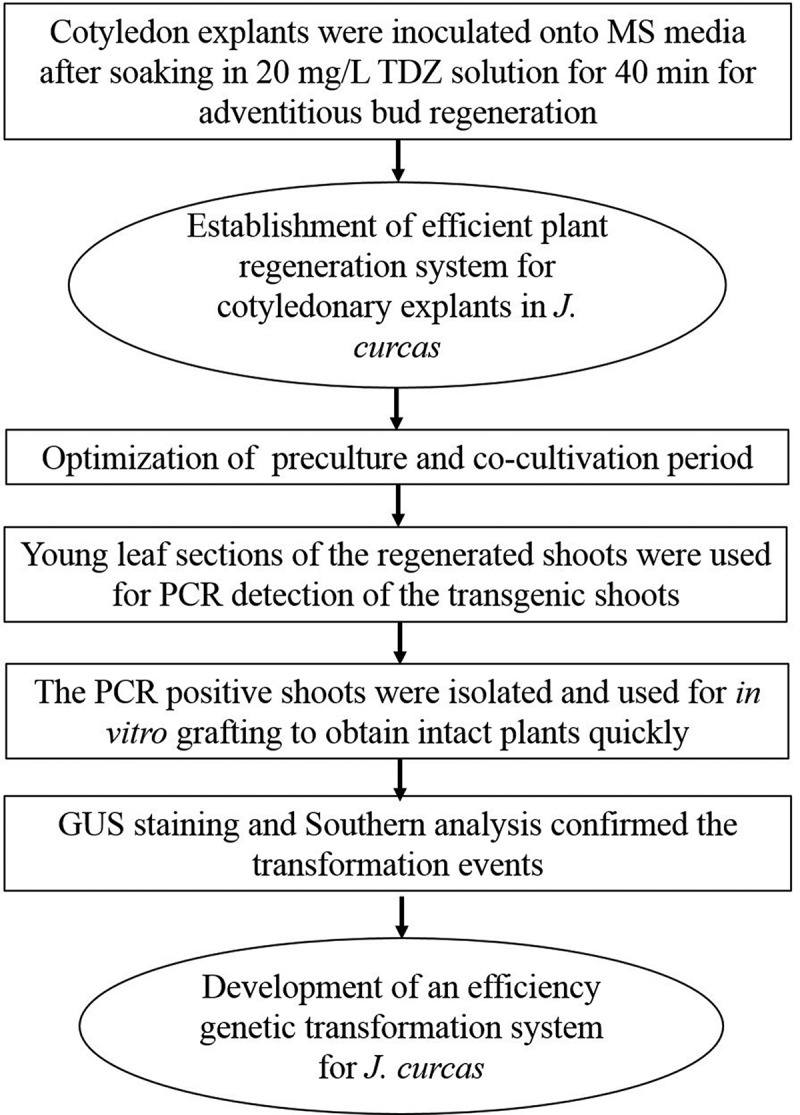

## Introduction

*Jatropha curcas* is a member of the genus *Jatropha* from *Euphorbiaceae* [1]. As a woody plant, *J. curcas* is mainly found in tropical and sub-tropical areas. The seed oil of *J. curcas* is generally not suitable for human consumption and animal feeding on account of the existence of toxic substances such as saponins toxic proteins and phorbol myristate acetates [2]. The emulsion produced from *J. curcas* includes total alkaloids contents like Jatropha alkali, which are proved to contain medical components and applied to the extraction of pharmaceutical compounds and the production of insecticides [3]. However, the best-known of this tree is because its seeds contain high oil content, and its seed oil could be suitably used in place of the traditional fossil fuels, and its methyl ester in the oil of seed could be used to manufacture biological diesel oil [4]. Therefore, the breeding of *J. curcas* has aroused, causing widespread concern around the world.

For the sake of satisfying the growing requirements for the applications of biodiesel, feedstock, and medicine, it is essential to cultivate improved *J. curcas* varieties. With the development of biotechnology vegetali, as well as the publication of the drafting whole genome sequences, a breeding improvement especially concerning oil content and seed yield, will be of importance for researchers through a transgenic strategy [1,5]. However, an important prerequisite for cultivating elite *J. curcas* varieties is to better understand the correlated metabolic pathways and the underlying genetic regulators. For most of the plant species, a transformation based on plant tissue culture is important for the above mentioned transgenic breeding and functional genomics studies.

*Agrobacterium tumefaciens*-mediated transformation is the preferential means in plant gene transformation and the effective means of plant genetic improvement. To apply the *Agrobacterium*-mediated genetic transformation approach to jatropha improvement more rapidly and efficiently, establishing the regeneration system of plant tissue culture is the precondition for plant gene transformation. As previously reported, the regenerated adventitious buds had been acquired smoothly from various types of explants in *J. curcas*, such as petioles, hypocotyls, leaves, epicotyls, and cotyledons, but the periods of obtaining regenerated plants were relatively long (90–140 days) [6–8]. A series of works have described an efficient and reproducible protocol in which the various explants were treated in high concentrations (5–120 mg/L) solutions of cytokinins (CTKs) such as 6-benzyl aminopurine (6-BA) and thidiazuron (TDZ) for short periods (5–80 min) in order to induce adventitious buds regeneration of soybean [9] and jatropha [10,11]. Our previous studies indicate that TDZ is a high-performance growth regulator for plant generation of *J. curcas*. After treatment with high concentrations of TDZ different, different kinds of petiole explants show significantly improved regeneration results compared with conventional culture methods [10,11].

Up to now, the explants from *J. curcas* such as leaves, cotyledons, hypocotyls, embryonic axes, and petioles have been used to genetically transform via *Agrobacterium tumefaciens*, and the plants transformed with genes were obtained by means of firsthand or indirect organogenesis [12–16]. However, the periods for obtaining transformed plants were still too long (90–160 days).

In the present study, one resultful plant regeneration method for *J. curcas* via making use of the highly concentrated TDZ solution to process the cotyledon explants was set up finally. Based on this method, genetically modified jatropha buds were gained conclusively via *Agrobacterium*-mediated genetic transformation, and transgenic jatropha plantlets were finally acquired by *in vitro* grafting method within 65 days.

## Materials and methods

### Plant material and culture conditions

The seeds of the M-19 genotype of *J. curcas* were gained from the planting base of South China Agricultural University in Haikou, Hainan Province, China. Seeds were dealt with 2% sodium hypochlorite solution, as mentioned as our previous report [10]. The immature embryos were obtained from jatropha seeds and inoculated onto MS (Murashige and Skoog) medium [17] without any hormone. Cotyledon explants were obtained from jatropha seedlings with 5 days of culture and then incised into 5 × 5 mm pieces.

Coincident conditions of tissue culture were adopted for all the experiments. Fundamental MS medium was applied to cultivate explants in this study. There was 2.5% sucrose added into all the mediums, which were regulated pH to 5.8–6.0 through adding 1 mol/l NaOH solution. While 0.7% agar was added into the mediums before being autoclaved for 20 min at 0.1 Mpa and 121°C. The explants were placed in a culture room with the temperature at 25 ± 1°C under a 12 h photoperiod. All the plant growth regulators used in the present study were bought directly from Sigma corporation (Sigma, California, America).

### Preparation of thidiazuron (TDZ) treating solution

TDZ (Sigma, USA) was dissolved first into 1 mol/L NaOH solution. The TDZ solution was diluted with deionized water to acquire different concentrations as follows: 0, 5, 10, 20, 30, 60, 120 mg/L. The pH value of those TDZ solution was readjusted with 1 mol/L HCl to 5.8–6.0. Furthermore, the TDZ solution was sterilized by passing through a filter having a pore size of 0.22 micron before using it.

### Soaking treatment for cotyledon explants with TDZ solution before regeneration culture

TDZ solutions with diverse concentrations (0, 5, 10, 20, 30, 60, 120 mg/L) were prepared as described previously [10]. Cotyledon explants were dipped into TDZ solution at above concentrations respectively for various times (0, 5, 10, 20, 40, 60, 80 min). Whereafter, the treated explants were inoculated on aseptic paper to eliminate excess water. In order to induce regeneration of adventitious shoots, cotyledon explants were placed onto MS medium without any hormone after soaking in TDZ solution at disparate concentrations for different times. As controls, cotyledon explants were also disposed with traditional ways and inoculated onto MS medium adding various TDZ concentrations (0, 0.1, 0.3, 0.6, 1.2 mg/L) as described previously [18,19]. The regeneration effect was evaluated after 30 days of culture.

### Elongation of the adventitious buds

In order to accelerate the extension of adventitious shoots, the regenerated adventitious buds with maternal tissues were moved onto MS medium contained 0.2 mg/L KT (Kinetin), 0.25 mg/L IAA (Indoleacetic acid), 0.4 mg/L GA_3_ (Gibberellin), and 0.5 mg/L 6-BA as in previous study [10]. The height of extended shoots was noted down and analyzed after 15 days of culture.

### Agrobacterium strains and binary vectors

For establishing the method of *Agrobacterium tumefaciens*-mediated transformation adopting cotyledon explants from *J. curcus*, the GUS reporter system was used for transformation and the following detection of transgenic shoots as described [20]. In brief, a modified pCAMBIA1300 vector named p1300G with the 35S promoter of *Cauliflower mosaic virus* (CaMV 35S), the β-glucuronidase (GUS) gene, the selectable marker gene for hygromycin phosphotransferase (Hpt), and the terminator of nopaline synthase (NOS). The total size of p1300G plasmid was 11.6 kb, and then it was introduced into *Agrobacterium* strain EHA105 using the method of liquid nitrogen freezing and thawing [21]. One clone of the transformed EHA105 bacteria was applied to transplanted into liquid YEB (Yeast Extract Mannitol Broth) media supplemented with 1 g/L yeast extract, 5 g/L peptone, 5 g/L beef extract, 5 g/L sucrose, 0.04 g/L MgSO_4_ · 7H_2_O, 100 mg/L kanamycin and 34 mg/L chloramphenicol (pH = 7.0) [22]. The bacteria were cultured at 28 degrees Celsius for the night. Bacterial cultures were obtained via centrifuges at the speed of 4000rpm for 10 min at 25 degrees Celsius. The centrifugal sediments were re-suspended by utilizing of 20 mL liquid MS media supplemented with 2 g/L inositol and 100 mg/L acetosyringone (AS) (pH = 5.8–6.0). Moreover, the optical density (OD_600_) value of the bacterial solution was regulated to the range of 0.1–0.2 before the genetic transformation.

### Transformation protocol of J. curcus

The cotyledons were placed onto preculture media (MS medium without any phytohormones) for different durations (0–4 days) after soaked into TDZ solution at 20 mg/L for 40 min and then infected with the solution of *Agrobacterium tumefaciens* for 30 s. The explants were then moved to the cocultivation media (MS media with 2 g/L inositol, 7 g/L agar, and 100 mg/L AS) and co-cultured for different times (0–6 days) at 28°C in the darkness. Thereafter co-cultivation, the processed explants were inoculated on regeneration and screening medium (MS media supplemented with 200 mg/L carbenicillin, 200 mg/L cefotaxime, 1.5 mg/L hygromycin, 2 g/L inositol, 7 g/L agar and 100 mg/L AS) to obtain adventitious buds for 30 days, and then the explants with regenerated buds were moved onto shoot bud elongation medium (MS media contained 0.4 mg/L GA_3_, 0.2 mg/L KT, 0.5 mg/L BA, 0.25 mg/L IAA, 1.5 mg/L hygromycin, 200 mg/L carbenicillin, 200 mg/L cefotaxime, 2 g/L inositol, and 7 g/L agar) for 15 days.

### PCR detection and in vitro grafting

Genomic DNA (Deoxyribonucleic acid) of potential transgenic buds and wild type control were isolated directly from the leaf material (cut into julienne with the width about 2 mm) by using the MightyAmp™ DNA Polymerase Ver.2 Kit (Takara, Tokyo, Japan). In order to test whether the selectable marker gene *Hpt* exists in elongated buds, PCR (Polymerase chain reaction) amplification of a 748-bp fragment of Hpt reporter gene was cloned with the primers of hptF (5ʹ-CATCGAAATTGCCGTCAACC-3ʹ) and hptR (5ʹ-GCTTTCAGCTTCGATGTAGG-3ʹ) to select putative transgenic bud by PCR amplifier (Bio-Rad, California, America). The PCR reactions were implemented in the volume of 20 μL, and the conditions and parameters of PCR reaction were as described below: 2 min at 98°C, followed by 30 cycles of 10 s at 98°, 15 s at 60°, and 50 s at 68°C, and followed by a final extension of 10 min at 68°C. Plasmid DNA of p1300G was treated as the positive control, while the DNA from the non-transgenic plant was used as the negative control. The PCR products were distinguished by 1.0% (w/v) agarose electrophoresis and viewed via the method of ethidium bromide staining under the excitation of ultraviolet (UV) light [13].

Next, the PCR-detected *Hpt* positive shoots were cut off treated as ingraftment scions, and then hypocotyls carrying with radical (length of 0.2 cm) from aseptic seedlings in 5 days of wild type *J. curcas* served as stocks. The protocol of *in vitro* grafting was implemented, as mentioned earlier (Liu et al. 2016). The upside of the rootstock was cleaved about 5 mm depth in the middle vertically downward, and the bottom of the scion was cut into wedge-shaped. The *in vitro* grafting could be completed by inserting the prepared scion carefully into the middle of the incision of rootstock [11]. After performing sterile micro-grafting, the graft plants were placed into 1/2 MS media contained 2 mg/L Gln and 0.3 mg/L IBA to enhance growth for about 20 days to obtain intact plants.

### Histochemical β-glucuronidase (GUS) assay

Histochemical staining of GUS activity was implemented on the basis of the protocol reported by Jefferson [20]. Leaves obtained from hygromycin-resistant shoots and wild type plants were placed into the assay solution (1 mg/L X-gluc, 0.16 mg/L K_3_Fe(CN)_6_, 0.21 mg/L K_4_Fe(CN)_6_ · 3H_2_O, 0.5% (w/v) Triton X-100 and 16.3 mg/L sodium phosphate, pH = 7.0) (Solarbio, Beijing, China) at the temperature of 37°C for the night. After staining treatment, the leaves were rinsed with 70% (w/v) ethanol solution for 3–5 times to get rid of redundant dyestuffs.

### Southern blot analysis

The genome DNA of *J. curcas* was obtained from tender leaves of individual plants via using Plant Genomic DNA Extraction Kit (Deaou, Guangzhou, China) in accordance with the manufacturer’s instruction. Hpt transgene detection by means of Southern blot was implemented as earlier procedures [23]. In order to accomplish Southern blot analysis, 8 μg of genomic DNA from transformants was digested with the restriction endonuclease *Hin*d III, distinguished by 0.8% (w/v) agarose electrophoresis, and moved to Hybond N^+^ nylon membranes (Amersham-Pharmacia, Connecticut, USA). The Hpt probe was labeled with digoxygenin (DIG) by PCR using the primers of hptF and hptR at the same procedure as mentioned above. These major steps of prehybridization, hybridization, and chemiluminescent detection of the blots were accomplished based on the kit instructions (Roche Diagnostics GmbH, Basel, Switzerland).

### Data analysis

All tests were adopted completely random and repeated 3 times, with 28–30 explants each time. Data processing was implemented with the help of SPSS 17.0 software, and then significant tests were conducted with Duncan multiple-range comparing, at p ≤ 5%. The quantitative data would be denoted with an average number ± standard deviation (SD).

### Results and discussion

*Establishment of stable and efficient plant regeneration system for cotyledonary explants in J. curcas*

**Figure 1.**
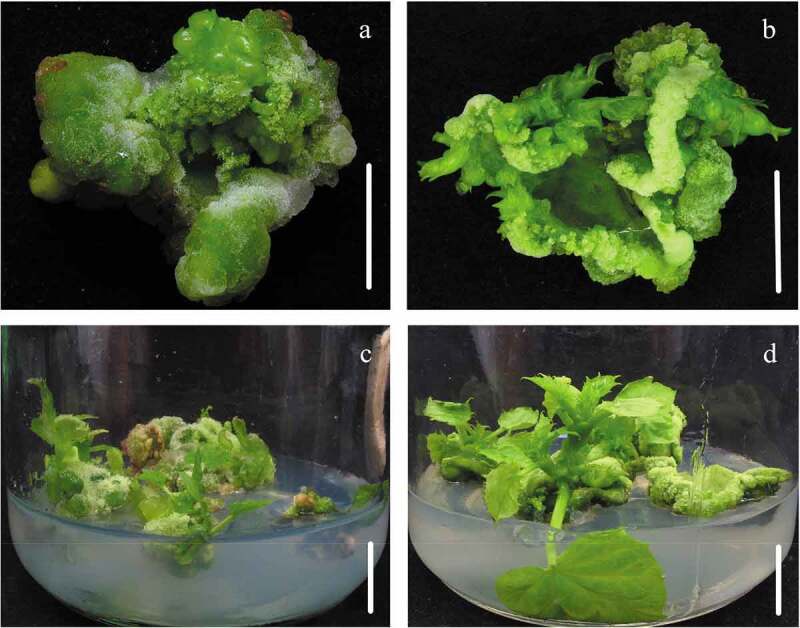
Induction of regenerative adventitious shoots from cotyledon explants in *J. curcas*. (a) Cotyledon explants were placed onto MS media supplemented with 0.6 mg/L TDZ after 30 days of culture, (b) Cotyledon explants were placed on MS media without plant hormone for 30 days of culture after being soaked into TDZ solution at the concentration of 20 mg/L for 40 min, (c) Extension of regenerative adventitious buds from traditional protocol, (d) Elongation of regenerative shoot-buds from treating explants with TDZ solution. Bars = 1 cm

TDZ was proved to be a plant growth mediation agent with the strong activity of cytokinin, had been put into use as a type of CTK (Cytokinin) to induce the adventitious shoot regeneration in numerous cases of plant tissue culture including *J. curcas* [19]. Application of traditional tissue culture methods for promoting regeneration of adventitious shoots, jatropha cotyledon explants were placed onto MS media with 0.05–2 mg/L TDZ [18,19]. We then detected the regeneration efficiency using the traditional method. Of the various concentrations of 0, 0.1, 0.3, 0.6, 1.2 mg/L TDZ used, the maximum rate of adventitious bud regeneration (42.39%) and the largest amount of buds (6.53) on each explant were acquired when using 0.6 mg/L TDZ (Table 1). Though the number of buds was not a few, a large amount of regenerate adventitious buds were completely thinness and undynamic (Figure 1a). While those buds from TDZ directly supplied in the regeneration mediums were difficult to elongation, the best elongation of buds was from the cotyledon explants being inoculated onto MS media with TDZ at a concentration of 0.6 mg/L, and it achieved the optimal average length of buds (1.09 cm) and the largest amount of shoots in excess of 2 pieces of leaves on each explant (1.20) (Table 1 and Figure 1c).

**Table 1.** Results of regeneration and elongation of adventitious shoots via a traditional protocol

TDZ concentration (mg/L) *	Regeneration of adventitious buds			Elongation of regeneration buds	
	Percentage of regeneration (%)	Number of buds per explant		The average length of buds (cm)	Number of shoots in excess of 2 pieces of leaves on every explant
0	0d**	0e		0 c	0 c
0.1	20.53 ± 3.21 c	3.51 ± 0.72d		0.74 ± 0.13b	0.91 ± 0.16ab
0.3	26.87 ± 1.31bc	4.65 ± 0.53 c		0.91 ± 0.09ab	1.11 ± 0.15a
0.6	42.39 ± 5.14a	6.53 ± 0.37a		1.09 ± 0.15a	1.20 ± 0.12a
1.2	31.50 ± 2.50b	5.79 ± 0.28b		0.64 ± 0.14b	0.81 ± 0.17b

*For analyzing the effectiveness on the elongation of the adventitious buds, maternal tissues going with regenerate adventitious buds from traditional protocol were moved onto MS media contained 0.25 mg/L IAA, 0.4 mg/L of GA_3_, 0.2 mg/L KT, and 0.5 mg/L 6-BA. **Different letters after the data indicated significant differences.

In our preceding research, dealing explants with high-concentrations TDZ solution could greatly increase the efficiency and quality of the bud regeneration of *J. curcus* petiole explants [10], the similar regeneration experiments were performed by using cotyledon explants obtaining from the seedlings after five days of culture. We first studied the bud induction efficiency on different dipped time periods of treating explants with TDZ solution before being inoculated onto MS media with no hormone, found that the induction of regenerate buds was affected obviously by dipped time periods (Table 2). The optimum results were gained when dealing explants with 20 mg/L TDZ solution for 40 min and the maximum rate of regeneration (86.44%) and the highest amount of adventitious buds on every explant (11.22) were acquired subsequently (Table 2 and Figure 1b). When the cotyledon explants were dealt with TDZ solution for time periods longer than 40 min, the rate of regeneration was reduced distinctly (Table 2).

**Table 2.** Influence of soaking cotyledon explants with TDZ solution at the concentration of 20 mg/L for a different time on the regeneration of adventitious buds in *J. curcas.*

Dipping time periods (min)	Percentage of regeneration (%)	Number of buds every explant
0	0e *	0e
5	45.11 ± 2.54d	5.25 ± 0.40d
10	59.64 ± 3.43 c	6.28 ± 0.37 cd
20	67.87 ± 3.26b	8.39 ± 0.39b
40 60	86.44 ± 4.82a 65.22 ± 2.35b	11.22 ± 0.51a 9.02 ± 0.43b
80	56.38 ± 2.69 c	7.00 ± 0.13 c

*Different letters after the data indicated significant differences.

Cotyledon explants were then dipped treatment with TDZ solution at different concentrations for 40 min before inducing adventitious bud. The induced effects of adventitious bud were affected visibly by TDZ solution (Table 3). When TDZ solution at the concentration of 20 mg/L was adopted, the optimal induction percentage of adventitious buds (86.44%) and the maximum amount of regenerative buds every explant (11.22) (Table 3 and Figure 1b) were gained respectively. The induction rate and the amount of regenerate adventitious buds every explant were concomitantly in accordance with the concentration of TDZ solution, when the concentration of TDZ solution was used at most 20 mg/L (Table 3). Nevertheless, while TDZ concentration was adopted in excess of 20 mg/L, the regeneration rate of adventitious buds was reduced visibly (Table 3). It’s clear that treatment with high concentrations of TDZ was performed more efficiently than the traditional method in order to induce regenerate adventitious buds from cotyledon. Moreover, the elongation results of the regenerated buds were better than the conventional method (Table 3). The optimal growth of adventitious buds was from the cotyledon explants inoculated onto MS medium after soaking processing with TDZ solution for 40 min at the concentration of 20 mg/L, and it achieved the first-rank of an average length of buds (1.82 cm) and the most amount of shoots in excess of 2 pieces of leaves on every explant (3.21) (Table 3 and Figure 1d).

**Table 3.** Results of soaking cotyledon explants with TDZ solution at different concentrations on adventitious bud induction in *J. curcas.*

TDZ solution concentration (mg/L) *	Induction of adventitious shoots			Elongation of regenerated buds**	
	Regeneration percentage (%)	Number of buds on every explant		The average length of buds (cm)	Number of shoots in excess of 2 pieces of leaves on every explant
0	0 f***	0 f		0	0
5	41.38 ± 3.12d	5.56 ± 0.38d		1.43 ± 0.11bc	2.38 ± 0.19b
10	57.23 ± 2.99 c	7.52 ± 0.29 c		1.58 ± 0.12b	2.94 ± 0.18a
20	86.44 ± 4.82a	11.22 ± 0.51a		1.82 ± 0.13a	3.21 ± 0.21a
30	68.37 ± 3.47b	9.49 ± 0.16b		1.65 ± 0.12ab	3.05 ± 0.24a
60	52.14 ± 2.58 c	7.69 ± 0.27 c		1.47 ± 0.16bc	2.37 ± 0.15b
120	25.64 ± 2.78e	3.77 ± 0.32e		1.21 ± 0.14 c	1.78 ± 0.17 c

*The whole explants were dipped treatment with TDZ solution for 40 min at various concentrations. ** For analyzing the effectiveness on the elongation of the adventitious buds, maternal tissues going with regenerate adventitious buds from traditional protocol were moved onto MS media contained 0.25 mg/L IAA, 0.4 mg/L of GA_3_, 0.2 mg/L KT, and 0.5 mg/L 6-BA. ***Different letters after the data indicated significant differences.

In the present study, the traditional protocols were demonstrated a relatively lower regeneration effect, and the regenerated buds were very difficult to farther elongation and development for cotyledon explants. Nevertheless, dipping treatment cotyledon explants with high-concentration TDZ solution for short-term times (HCST) before placing onto MS media with no hormone promoted the regeneration effect and generated the production of larger buds in contrast to the traditional method, and further culture found that those regenerative adventitious buds were extended readily, and similar results have been observed when using different kinds of petioles as explants in *J. curcas* [10,11], indicating that this regeneration procedure is probably suitable for all type of commonly used explants of *J. curcas*. Till now, different types of explants, including petioles from mature trees [10], petioles from seedlings for 20 days of culture and planted in soil [11], and *in vitro* cotyledons from 5-day-old seedlings in this study were used for the adventitious bud induction using this HSCT method based on TDZ. For the several types of explants, the best bud induction rate (91.36%) was gained from the petiole explants from 20-day-old-seedlings [11], and the petioles from mature trees had the worst induction results, i.e. 65.78% induction rate per explants [10]. The best induction results were obtained by using TDZ solution to deal with different petiole explants for 20 min at the concentration of 20 mg/L [10,11]. However, dipping treatment cotyledons with 20 mg/L TDZ for 40 min got the best induction results for cotyledon explants in this study, indicating that different explants may have different responses to TDZ.

TDZ is one type of cytokinins generally applied in tissue culture and plantlet regeneration of xylophyta [24]. Research shows that the production of adventitious bud regeneration from many kinds of plant explants can be induced by cytokinins such as TDZ [24,25]. Our research indicated that cotyledon explants might only need a short-term of cytokinin stimulation like a signal switch during induction of adventitious buds. Using the new protocol could gain higher quality regenerated buds (larger buds), which was consistent with our previous reports [9,11]. Cytokinins might not be necessary during the whole process of adventitious bud induction when the adventitious buds had been formed after cell division and differentiation, the exogenous cytokinins might not be needed; rather, the exogenous hormones existed for a long time in media might have suppression effect on the regeneration of adventitious buds. It has been reported in many books and scholarly journals that cytokinins can restrain the growth of root tip and inhibit root development.

#### Development of an efficient genetic transformation system for J. curcas

Based on this regeneration procedure, a protocol of *Agrobacterium tumefaciens*-mediated transformation for cotyledonary explants from *in vitro* 5-day-old seedlings had been established. In advance of co-culture with *Agrobacterium* liquid, cotyledonary explants were inoculated onto hormone-free MS media for some time (0 to 4 days). As shown in Table 4, the cotyledonary explants were infected with *Agrobacterium* solution immediately without passing through the preincubate and cultured onto the induction media of hygromycin-resistant buds, showed the lowest induction efficiency of hygromycin-resistant buds (5.13%) than those precultured ones, and cotyledonary explants precultured for 1 day showed the highest hygromycin-resistant bud induction efficiency (26.52%), and longer durations (2–4 days) resulted in lower induction efficiency (12.78–17.46%). In the end, one-day preculture was adopted in the *J. curcas* transformation system in this study (Table 4). As shown in Table 4, the optimum hygromycin-resistant bud induction efficiency (27.85%) was gained when the cotyledonary explants with *Agrobacterium* liquid were carried on co-culture for 2 days. Meanwhile, the induction effect (10.27–16.27%) was not enhanced by the extended cycle of co-culture. As a result, a 2-day co-culture in the dark was consistently implemented in the *J. curcas* transformation protocol in this study.

**Table 4.** Effect of preculture and co-cultivation period on regeneration efficiency of hygromycin-resistant buds (%) for cotyledonary explants of *J. curcas.*

Parameters	Culture time (d)	Induction percentage of hygromycin-resistant buds (%)
Preculture	0	5.13 ± 2.22d*
	1	26.52 ± 2.86a
	2	17.46 ± 1.31b
	4	12.78 ± 1.77 c
Co-cultivation period	0	2.57 ± 2.23d
	1	16.44 ± 1.89b
	2	27.85 ± 1.86a
	3	16.27 ± 2.13b
	6	10.27 ± 2.28 c

*Different letters after the data indicated significant differences.

**Figure 2.**
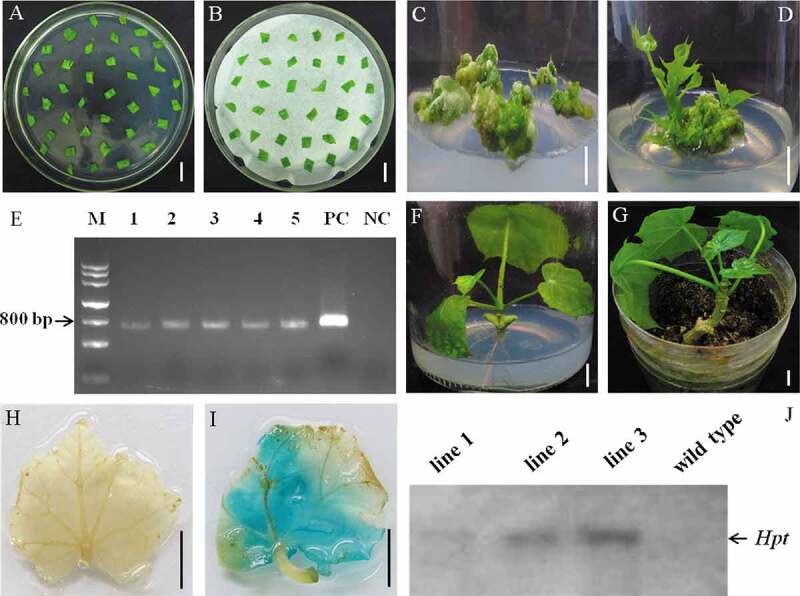
Establishment of genetic transformation system with adopting cotyledonary explants in *J. curcas* and identification of transgenic plants. (a) Cotyledons were placed onto preculture medium for 1 day after dealing explants with TDZ solution for 40 min at the concentration of 20 mg/L, (b) *Agrobacterium*-infected cotyledonary explants were placed onto a sheet of sterilized blotting paper on co-culture media for 2 days under dark culture, (c) Cotyledonary explants were moved onto MS medium supplemented with 200 mg/L carbenicillin, 200 mg/L cefotaxime and 1.5 mg/L hygromycin for 30 days of culture, (d) Maternal tissues with resistance buds were inoculated on MS media contained 0.4 mg/L GA_3_, 0.2 mg/L KT, 0.5 mg/L BA, 0.25 mg/L IAA, 1.5 mg/L hygromycin, 200 mg/L carbenicillin and 200 mg/L cefotaxime for the sake of elongation after 15 days of culture, (e) The amplification of PCR of the *Hpt* reporter gene in resistant buds (lines 1 to 5), M: DNA marker, PC: the plasmid positive control (p1300G), NC: untransformed negative control, (f) Grafting seedling was moved onto 1/2 MS media contained 2 mg/L Gln and 0.3 mg/L IBA at day 20, (g) Grafted plant was successfully lived well after being transplanted into soil, (h-i) GUS assay of non-transformed plantlet (h) and the transformed plant (i), (j) Results of Southern hybridization using the *Hin*d III to digest *J. curcas* genome DNA (about 8 μg for each sample) from transformed plants (lines 1 to 3) and control group. Bars = 1 cm

A protocol of *A. tumefaciens*-mediated transformation for cotyledonary explants of *J. curcas* was established according to the results mentioned above. The cotyledon explants were immersed into TDZ solution for 40 min at the concentration of 20 mg/L before placed in preincubate media (MS without any plant growth regulators) (Figure 2a). One day later, the above cotyledon explants were implemented *Agrobacterium* infection for 30 s followed by two days of co-culture on MS media with 1 mg/L AS (Figure 2b). After 2-day dark treatment, the explants were placed in adventitious bud induction, and screening media (MS medium contained 200 mg/L carbenicillin, 200 mg/L cefotaxime, and 1.5 mg/L hygromycin) (Figure 2c). After 30 days of culture, maternal tissues with resistance buds were moved onto the elongation medium to produce elongated resistant shoots for 15 days (Figure 2d). Before *in vitro* grafting, 2 mm in diameter of the young leaves of the elongated shoots was cut and used for PCR-based *Hpt* detection. Gene-specific PCR for *Hpt* selection marker gene was performed to select the putative transgenic shoots using the above leaf sections (diameter less than 2 mm) as a template. A PCR product with the expected length was tested in the transgenic plant lines, and this product could not be examined in no genetically modified plantlets (Figure 2e). PCR product was verified by sequencing. By applying the PCR-based determination system, a total of 46 potential transgenic buds were acquired from 134 cotyledon explants within 45 days, and the efficiency of gene transfection was 34.40% (Table 5). Our results presented in this study showed that this HSCT protocol might be widely applied to accomplish the establishment of *Agrobacterium tumefaciens*-mediated transformation.

**Table 5.** Results of *Agrobacteriuim*-mediated cotyledon transformation in *J. curcas.*

Experiment	Number of explants	Number of resistant buds	Number of elongated shoots with length ≥0.5 cm	PCR positive (%)
1	39	54	41	14 (35.90)
2	46	59	45	15 (32.61)
3	49	61	46	17 (34.69)
			The average value of PCR positive	34.40%

Then, the candidate transgenic shoots were isolated as scions and were grafted into the stocks of hypocotyls from 5 days old asepsis seedlings in wild type *J. curcas*. After accomplishing sterile micro-grafting, the grafting seedlings were all transferred to 1/2 MS media contained 2 mg/L Gln and 0.3 mg/L IBA to enhance the development of them, and intact plants with multiple leaves and adventitious roots can be obtained within 20 days. As demonstrated in Figure 2f and g, it was obvious that plentiful roots were germinated in the grafted plant, which was alive and well after being cultivated into the soil.

In this study, one kind of early PCR detection method to select the putative transgenic shoots was adopted. Young leaves from the elongated buds were cut into julienne with a width of about 2 mm were applied to carry out PCR reaction. Meanwhile, the genomic DNA of potential transgenic buds and wild type control were extracted directly from the leaf material by using the MightyAmp™ DNA Polymerase Ver.2 Kit. Nevertheless, the conventional approach of PCR detection is not implemented until obtaining complete potential transgenic plants in the end, and DNA is usually extracted from plant material by using the method of cetyltrimethylammonium bromide (CTAB) [26]. Furthermore, the buds with PCR positive result, even those short and small ones (length less than 2.5 cm), could be cut from their parent materials and considered as scions for *in vitro* grafting, so the specific procedure of bud elongation culture had been subtracted, and the period of obtaining transformed intact plants would be shortened.

Grafting is a universal protocol in gardening for propagating elite buds or shoots, has been applied to plant genetic improvement [15,27]. *In vitro* grafting is the biotechnology of grafting stocks with scions in sterile conditions. It combines tissue culture with grafting technology [28,29]. Micro-grafting techniques were used in the fields of recovery and multiplication of transgenic plants with failing to acquire a high success rate. For example, *in vitro* and *ex vitro* grafting techniques are applied to recover transgenic *Gossypium hirsutum* plants, which gain about 90% and 71.9% of successful rate separately [30]. Similarly, after using the *in vitro* shoot grafting in *Camellia sinensis* plants, 90% of the success rate is obtained subsequently [31]. In addition, the genetic stability of the grafted plants was the major focus during the recovery and multiplication of transgenic plants via grafting. The genetic stability of the grafted plants is estimated by utilizing the technology of randomly amplified polymorphic DNA (RAPD). The results show that 100% of the genetic stability rate is acquired between grafted plants and maternal plants [27]. By applying *in vitro* grating method, we gained the grafted plants quickly and successfully, even from those small shoots (longer than 0.5 cm). However, when adopting the conventional grafting method, only those shoots or buds with higher quality (2.5–5 cm in length) can be collected and used as scions, while the small and thin buds are not chosen and eliminated in the end [27,32]. Moreover, this method may overcome the difficulties in growing and rooting of the transgenic buds and be applicable in other woody plant species. Alternatively, the elongated shoots can be excised and cultured for rooting [10,11], which could eliminate the possible effect on the genetic instability of the grafted plants.

The expanded leaves from grafted plantlets and WT plantlets in the soil were tested via histochemical staining of the GUS gene. The strong blue color was detected from the leaf blade of the candidate transgenic shoot (Figure 2i), and no blue staining was observed from the non-transgenic plant leaf blade (Figure 2h), showing that the GUS fusion gene was expressed normally in *J. curcas*. Southern hybridization was also performed to verify the authentic transgenic plants. As shown in Figure 2j, the wild type plants without *Hpt* selection gene in the genome showed no hybridization band. The putative transgenic plant line 1 exhibited a weak hybridization band possibly caused by the low amount of genomic DNA or poor quality of DNA, and the other two regenerated plants (line 2 and line 3) showed relatively strong hybridization bands, indicating that *Hpt* gene had been integrated into the *J. curcas* genome and these two plants are transgenic plants (Figure 2j).

In the process of plant genetic transformation, the formation of callus and induction of adventitious bud regeneration are inhibited observably by selective agents such as hygromycin [33], kanamycin [16], herbicide [34], and so on. These selective agents do harm to the development and growth of plant cells, even the transformed ones, causing transformed cells regenerated unhealthy buds and resulting in a long period of recovery of transgenic plants prepared for transplantation from *in vitro* to the soil [16,33,34]. In order to further improve the efficiency of *Agrobacterium*-mediated genetic transformation of *J. curcas*, the method of selectable marker-free is one of the valuable research directions [35]. Because the screening marker genes on the vectors will be removed after transformation, those selective agents may not be used in the process of plant transgenic. This approach has been successfully developed for several plant species, including *Oryza sativa* [36], *Solanum tuberosum* [37], and *Vitis vinifera* [38]. The selectable marker-free method may be adapted and applied to the genetic transformation of *J. curcas* in future research.

In summary, a high-performance plant reproduction method and *A. tumefaciens*-mediated transformation procedure were built up by adopting cotyledon explants from *in vitro* 5-day seedlings of *J. curcas*, which have the capacity to achieve the genetic improvement and transgenic breeding of *J. curcas* cultivars.

## Conclusions

In this study, an efficient plant regeneration method and genetic transformation system of *J. curcas* was established to improve the effect of *Agrobacterium*-mediated genetic transformation, which were optimized from the following aspects. (1) An efficient method of adventitious bud regeneration from cotyledon explant was developed, better quality and more quantity of adventitious buds had been gained in a relatively short time (within 30 d) with no additional requirement of bud propagation culture, so the regeneration method was well applied to *Agrobacterium*-mediated genetic transformation of *J. curcas*. (2) The detection method for marker genes had been carried out in advance in the present study, small leaf material (diameter less than 2 mm) from resistant shoots could be used directly for PCR detection of screening marker genes after elongation culture of resistant buds, so the period for acquiring positive plants was observably shortened. Nevertheless, PCR detection with the traditional method would not be performed until the complete resistant plant was obtained. (3) The time of preculture and co-culture was optimized to enhance the efficiency of genetic transformation. (4) One way of *in vitro* grating method with using small transformed buds as scions was established, and the utilization efficiency of transformed buds was improved subsequently. By using the methods of this study, the period of obtaining complete transgenic Jatropha plants was markedly reduced. Furthermore, the results presented in this paper will be applicable for studying genes function and breeding new varieties in *J. curcas*.

## Article highlights

1. A new method for inducing adventitious bud regeneration from cotyledon was established.

2. Adventitious bud had been acquired within 30 d without extra bud multiplication culture.

3. The PCR detection approach for marker genes had been accomplished early in this study.

4. The period of preculture and co-culture had been optimized.

5. *In vitro* grating method was brought in the genetic transformation of *J. curcas*.
